# Nation-scale adoption of new medicines by doctors: an application of the Bass diffusion model

**DOI:** 10.1186/1472-6963-12-248

**Published:** 2012-08-10

**Authors:** Adam G Dunn, Jeffrey Braithwaite, Blanca Gallego, Richard O Day, William Runciman, Enrico Coiera

**Affiliations:** 1 Centre for Health Informatics, Australian Institute of Health Innovation, University of New South Wales, Sydney, 2052, NSW, Australia; 2 Centre for Clinical Governance Research in Health, Australian Institute of Health Innovation, University of New South Wales, Sydney, Australia; 3 Department of Clinical Pharmacology, St Vincent’s Hospital, University of New South Wales, Sydney, Australia; 4 School of Psychology, Social Work and Social Policy, University of South, Sydney, Australia

**Keywords:** Adoption, Diffusion of innovation, Decision-making, Prescribing behaviour, Australia, Evidence-based practice

## Abstract

**Background:**

The adoption of new medicines is influenced by a complex set of social processes that have been widely examined in terms of individual prescribers’ information-seeking and decision-making behaviour. However, quantitative, population-wide analyses of how long it takes for new healthcare practices to become part of mainstream practice are rare.

**Methods:**

We applied a Bass diffusion model to monthly prescription volumes of 103 often-prescribed drugs in Australia (monthly time series data totalling 803 million prescriptions between 1992 and 2010), to determine the distribution of adoption rates. Our aim was to test the utility of applying the Bass diffusion model to national-scale prescribing volumes.

**Results:**

The Bass diffusion model was fitted to the adoption of a broad cross-section of drugs using national monthly prescription volumes from Australia (median R^2^ = 0.97, interquartile range 0.95 to 0.99). The median time to adoption was 8.2 years (IQR 4.9 to 12.1). The model distinguished two classes of prescribing patterns – those where adoption appeared to be driven mostly by external forces (19 drugs) and those driven mostly by social contagion (84 drugs). Those driven more prominently by internal forces were found to have shorter adoption times (p = 0.02 in a non-parametric analysis of variance by ranks).

**Conclusion:**

The Bass diffusion model may be used to retrospectively represent the patterns of adoption exhibited in prescription volumes in Australia, and distinguishes between adoption driven primarily by external forces such as regulation, or internal forces such as social contagion. The eight-year delay between the introduction of a new medicine and the adoption of the prescribing practice suggests the presence of system inertia in Australian prescribing practices.

## Background

Problematic uptake of evidence into clinical practice is seen as a fundamental problem in delivering quality and safety in healthcare – both because the adoption of new evidence is seen as being too slow [1-3], and because factors other than evidence appear to have a strong influence over clinical decision-making, particularly for prescription medicines. Since the seminal work on the adoption of new medicines was published in the 1960s [4-7], relatively little attention has been paid to measuring population-wide adoption of prescription drugs in healthcare. The intervening period has seen dramatic increases in the volume of published evidence [8,9], the rise of me-too drugs [10], and increasing concerns about the confluence of clinical evidence and marketing [11-15]. Given these changes, a renewed interest in measuring adoption and understanding the factors that contribute to the adoption of new medicines into clinical practice is warranted.

For individual clinicians outside of acute care settings, decision-making is known to be driven by exposure to factors that include pharmaceutical company marketing [16], clinical practice guidelines and other forms of synthesised evidence, subsidisation, and the advice of colleagues and perceived local consensus [17-23]. Using individual choices to replicate or predict adoption at population-wide levels has been attempted using agent-based models [24,25].

At population-wide scales, investigations into patterns of adoption have measured adoption times using a variety of models [26,27]. Cohen [28] looked for differences in adoption patterns for pioneers (first-in-class drugs) versus followers (me-too drugs), without finding a general explanation. Yet others have examined the effects of changing evidence on practices that are already embedded in mainstream practice [29,30], and the reasons for differences in prescribing practices between countries [31]. Diffusion of innovation theory includes a set of models that aim to represent or predict the adoption patterns of new technology, products or ideas [32]. Mathematical models representing diffusion of innovation have been extensively reviewed [33-35]. These models are used to predict market penetration and adoption rate by analogously comparing them across products and environments, as well as forecasting market penetration and adoption rate using early time series data – with varied success.

**Figure 1:**
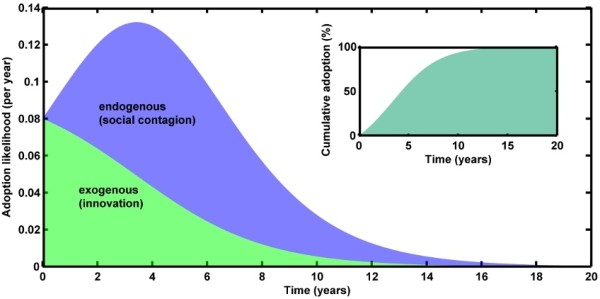
**The characteristic adoption curve as described by the Bass diffusion model.** The contributions to the S-shaped cumulative adoption curve (inset) comprise the internal and external factors. In this artificial example created using typical values for *p* and *q*, the adoption reaches 95 % of the population in approximately 12 years.

The Bass diffusion model [36-40] is the most common mathematical representation of diffusive adoption, describing the number of new adopters per unit time by the additive effects of external (designated by a parameter *p* in the model) and internal (designated by a parameter *q* in the model) forces (Figure 1), which may be useful when examining the factors contributing to an adoption rate. The Bass diffusion model has been demonstrated as a reliable model for hundreds of new innovations, often repeated in multiple marketplaces (such as different countries), and the consistency of the model has been examined in several meta-analyses and reviews [35,40,41].

The aims of the present study were to evaluate the Bass diffusion model in its ability to represent the prescription patterns of medicines introduced in Australia. A secondary aim was to provide descriptive statistics for adoption times of subsidised medicines in Australia.

## Methods

### Study data

Monthly prescription volumes for 103 drugs were retrieved from January 1992 to December, 2009 from aggregated, routinely collected data from the Drug Utilisation Database maintained by the Drug Utilisation Subcommittee (DUSC) of the Australian Pharmaceutical Benefits Advisory Committee (PBAC). Ethics approval was not required. Where a medicine was prescribed in more than one form, the data were aggregated into a single time series. Only those drugs with first recorded prescriptions after January 1992 were included in the analysis. The drugs were chosen to be representative of the set of drugs that are commonly-prescribed in Australia, other than over-the-counter drugs. The set is distributed across 11 of the 14 anatomical main groups, 33 different therapeutic subgroups including 65 pharmacological subgroups in the Anatomical Therapeutic Chemical classification. Note that in cases where a drug was represented in more than one group, we assigned it to a single group associated with the most common reason for prescription.

Importantly, some of the drugs included in the set have been shown to be unsafe or not cost-effective in relation to existing drugs following new published evidence within the time frame of the study, which may have a delayed or reduced effect on prescribing practices. The most prominent are rosiglitazone and rofecoxib, which were later withdrawn or restricted around the world [42-45]. In other cases, newly-introduced drugs provided cost reductions or slight gains in efficacy or safety rather than new molecular entities designed to fill an unmet need in the therapeutic class [46,47]. These characteristics are not considered in the analysis.

### Study Design

**Figure 2:**
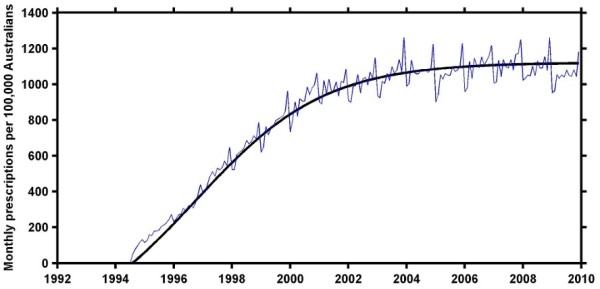
**The pattern of adoption for sertraline in Australia.** The pattern of adoption for sertraline is given by the raw monthly prescription volumes indicating the seasonal and safety net fluctuations (blue), and the Bass diffusion model estimate of the adoption over time (black). The adoption period (to 90 % of saturation) was between mid-1994 and the middle of 2003.

Raw monthly prescription volumes exhibit seasonal and safety net fluctuations [27], so they are smoothed (using a moving average over non-zero values) and then normalised by the population growth in Australia to give the number of prescriptions per 100,000 Australians. The smoothed and normalised monthly prescription volumes were used to represent the cumulative percentage of adoption by fitting them to the Bass diffusion model (Figure 2). The model was fitted using a non-linear least squares analysis from Matlab® 7.11.1 (The MathWorks, Natick, MA). The resulting values for *p* and *q* were used to classify the adoptions as either external-dominant (*p* > *q*) or internal-dominant (*p* < *q*), following van den Bulte & Stremersch [40].

The adoption time of a practice is defined to be the number of months between the first recorded prescription and the modelled estimate of 95% of the maximum monthly prescription rate (chosen arbitrarily to represent near-saturation as the model asymptotes at the maximum). In searching for factors associated with fast or slow adoption, correlations between the adoption time and specific factors that might be expected to influence adoption were considered. Firstly, the medicines were categorised by anatomical groups (via the Anatomical Therapeutic Chemical classification) and differences in adoption times were considered across the larger groups. Secondly, the adoptions were placed in two groups representing the strength of internal and external forces – those in which external forces were dominant, and those for which internal forces were dominant. In both cases, the statistical comparisons were performed using a Kruskal-Wallis test – a non-parametric analysis of variance across two or more groups. All tests were performed using Matlab® 7.11.1 (The MathWorks, Natick, MA).

A single class of drugs was used to illustrate order of market entry and system inertia. The drug class chosen was HMG CoA reductase inhibitors (statins). High cholesterol is the third largest contributor of risk to mortality worldwide behind smoking and high blood pressure [48]. Statins are the most common pharmacological treatments for the condition, and are recommended as best practice following lifestyle changes [49]. The cross-section of drugs included four statins, totalling 17.3 million prescriptions in Australia in 2009, and these were illustrated alongside simvastatin, which was introduced prior to 1992.

## Results

### Patterns of adoption

The Bass diffusion model was fitted to the prescription volumes of 103 medicines that were introduced between 1992 and 2009 (Table 1). After fitting the model using a non-linear least squares analysis, the median adjusted R^2^ value for the 103 adoptions is 0.97 with an inter-quartile range of 0.95 to 0.99, indicating an accurate fit. These values are similar to those reported for other products outside of healthcare delivery [50]. The results indicate that the median estimated adoption time is 8.2 years, with a relatively wide inter-quartile range of 4.9 years to 12.1 years.

**Table 1:** Adoption times for medicines adopted in Australia, by drug class

**Medicine (INN), by Anatomical main group (ATC)**	**Month of the first subsidised prescription**	**Maximum monthly prescriptions (per 100,000 Australians)**	**p/q ratio from Bass diffusion model**	**Model fit for Bass diffusion model (R**^2^**)**	**Modelled Adoption time (years)**
**Alimentary tract and metabolism**					
Esomeprazole	Mar 2002	2755	5.87×10^-1^	0.990	8.67
Clarithromycin*	Dec 1998	384	1.50×10^9^	0.865	14.08
Granisetron	Dec 2003	45	3.30×10^-2^	0.986	5.58
Ursodeoxycholic acid*	Jun 2000	13	8.50×10^9^	0.948	9.75
Balsalazide	Mar 2005	9	1.08×10^-1^	0.995	4.58
Insulin lispro	Jun 1996	30	4.52×10^-1^	0.973	6.75
Insulin glargine	May 2006	113	5.57×10^-1^	0.961	5.50
Glimepiride	Jun 2000	263	3.40×10^-2^	0.981	2.42
Rosiglitazone	Jun 2003	228	3.40×10^-2^	0.969	4.17
Pioglitazone	Jun 2003	226	1.21×10^-1^	0.912	8.83
Acarbose	Jun 1997	38	6.20×10^-2^	0.991	2.25
**Blood and blood forming organs**					
Enoxaparin	Nov 1993	123	4.00×10^-3^	0.990	14.42
Clopidogrel	Apr 1999	1327	2.69×10^-1^	0.994	11.33
Ticlopidine	Nov 1992	15	1.90×10^-2^	0.976	6.08
Dipyridamole	Mar 1999	74	1.10×10^-2^	0.962	1.17
Abciximab	Dec 1995	2	5.00×10^-2^	0.965	5.83
Tirofiban	Jun 1999	1	3.37×10^-1^	0.979	4.92
**Cardiovascular system**					
Nicorandil	Sep 1997	110	6.97×10^-1^	0.979	13.58
Eplerenone	Sep 2005	5	1.19×10^-1^	0.986	5.00
Bisoprolol	Mar 2002	211	2.80×10^-2^	0.983	9.08
Carvedilol	Dec 1997	256	1.39×10^-1^	0.991	9.50
Amlodipine*	Mar 1993	1378	1.86×10^9^	0.863	12.92
Lisinopril	Apr 1992	744	1.79×10^-1^	0.992	4.92
Perindopril	Mar 1992	3174	4.60×10^-2^	0.991	18.08
Ramipril	Apr 1992	1550	5.60×10^-2^	0.976	14.83
Quinapril	Sep 1992	453	8.00×10^-3^	0.953	7.25
Fosinopril*	Apr 1992	491	2.45	0.933	8.75
Trandolapril	Nov 1994	424	6.90×10^-2^	0.980	4.58
Eprosartan	Apr 1999	98	3.50×10^-2^	0.983	6.25
Irbesartan	Dec 1997	3333	6.51×10^-1^	0.992	9.33
Candesartan	Sep 1998	1640	1.02×10^-1^	0.995	11.83
Telmisartan	Jun 1999	1535	1.07×10^-1^	0.969	12.25
Pravastatin	Jan 1993	895	9.20×10^-2^	0.991	10.58
Fluvastatin	Sep 1995	223	2.80×10^-2^	0.952	1.83
Atorvastatin	Sep 1997	5096	8.67×10^-1^	0.987	13.42
Rosuvastatin	Jun 2006	1967	3.60×10^-2^	0.990	3.83
Fenofibrate	Mar 2004	250	3.00×10^-2^	0.991	6.00
Ezetimibe*	Mar 2004	419	1.30	0.974	6.17
**Dermatologicals**					
Fluconazole*	May 1992	11	2.83×10^8^	0.909	23.33
Imiquimod	Nov 2005	14	1.33×10^-1^	0.952	1.42
Fluticasone	Mar 1995	552	1.20×10^-2^	0.979	5.17
Tacrolimus	Mar 2003	6	2.46×10^-1^	0.967	9.92
Finasteride*	Jun 1995	25	1.71	0.838	18.50
**Genito urinary system and sex hormones**					
Raloxifene	May 1999	153	4.80×10^-2^	0.994	2.75
Alprostadil	Jun 1995	64	2.00×10^-3^	0.989	2.42
**Antiinfectives for systemic use**					
Roxithromycin	Jun 1992	1244	7.50×10^-1^	0.972	5.33
Azithromycin	Jan 1995	47	1.26×10^-1^	0.916	17.17
Itraconazole*	Jun 1997	3	1.94×10^8^	0.955	8.75
Famciclovir*	Jun 1995	64	4.79×10^11^	0.902	17.08
Valaciclovir	Mar 1996	160	1.54×10^-1^	0.997	13.50
**Antineoplastic and immunomodulating agents**					
Temozolomide	Sep 1999	5	5.98×10^-1^	0.910	12.33
Gemcitabine	Dec 1995	9	2.08×10^-1^	0.974	8.50
Capecitabine*	Jun 1999	9	2.22	0.928	8.58
Vinorelbine	Jun 1998	1	4.10×10^-2^	0.980	2.00
Paclitaxel	May 1994	9	2.96×10^-1^	0.942	17.92
Docetaxel	Mar 1996	9	5.39×10^-1^	0.850	19.25
Oxaliplatin	Jun 2001	8	4.46×10^-1^	0.971	5.00
Rituximab	Sep 1998	18	1.85×10^-1^	0.983	10.50
Imatinib	Jul 2001	9	1.70×10^-2^	0.976	1.92
Irinotecan	Dec 1999	5	8.00×10^-3^	0.865	1.08
Nilutamide	Dec 1996	2	3.10×10^-2^	0.977	1.92
Anastrozole	Mar 1997	89	2.00×10^-3^	0.982	12.42
Letrozole	Dec 1997	51	6.00×10^-2^	0.928	15.42
Exemestane	Aug 2000	9	2.03×10^-1^	0.921	11.67
Interferon beta1a*	Sep 1998	16	2.87×10^7^	0.977	9.75
Interferon alfa2a	Jul 1992	1	1.20×10^-2^	0.900	8.33
Leflunomide*	Sep 1999	78	2.87×10^9^	0.948	12.58
Etanercept	Mar 2003	27	8.60×10^-2^	0.989	7.58
Adalimumab	Dec 2003	36	1.90×10^-2^	0.980	6.92
**Musculo-skeletal system**					
Rofecoxib	Jun 2000	1281	1.40×10^-2^	0.963	1.75
Celecoxib	Jun 1999	1806	2.00×10^-3^	0.981	1.58
Alendronic acid	Jun 1996	955	9.00×10^-3^	0.991	8.25
Risedronic acid	Sep 2000	583	6.20×10^-2^	0.993	8.42
**Nervous system**					
Fentanyl	Mar 1999	203	6.00×10^-3^	0.910	12.25
Tramadol	Apr 1999	954	1.10×10^-2^	0.962	4.33
Oxcarbazepine*	May 1999	6	3.84	0.955	8.42
Lamotrigine	Jul 1994	146	7.11×10^-1^	0.984	17.17
Topiramate	Mar 1997	87	1.80×10^-1^	0.921	18.25
Gabapentin	Jul 1994	75	8.10×10^-2^	0.974	14.67
Flupentixol	Mar 1994	8	4.99×10^-1^	0.988	5.08
Zuclopenthixol	Jun 1996	10	1.21×10^-1^	0.956	6.83
Olanzapine*	Mar 1997	391	1.87	0.961	11.08
Quetiapine	Jun 2000	262	5.90×10^-2^	0.950	11.75
Amisulpride	Mar 2002	36	8.60×10^-2^	0.952	2.42
Risperidone	Sep 1994	268	9.10×10^-2^	0.927	17.67
Aripiprazole*	Dec 2003	47	5.50	0.975	5.50
Citalopram	Sep 1997	757	6.30×10^-2^	0.994	5.50
Paroxetine	Mar 1994	581	1.33×10^-1^	0.996	5.92
Sertraline	Mar 1994	1181	3.55×10^-1^	0.985	9.67
Fluvoxamine	Mar 1997	183	2.21×10^-1^	0.997	8.00
Venlafaxine	Mar 1996	1167	1.15×10^-1^	0.990	12.83
Methylphenidate	Feb 2005	156	6.50×10^-2^	0.992	3.92
Donepezil	Apr 1999	102	3.83×10^-1^	0.955	6.75
Rivastigmine	Mar 2000	13	1.00×10^-3^	0.946	1.42
Galantamine	Jun 2001	47	1.10×10^-1^	0.996	4.92
Acamprosate	Jun 1999	12	2.40×10^-2^	0.944	0.92
Riluzole*	Feb 2003	3	3.49×10^5^	0.975	6.08
**Respiratory system**					
Nedocromil	Nov 1994	115	5.00×10^-2^	0.986	2.08
Salmeterol	Sep 1994	1362	1.10×10^-2^	0.944	10.00
Formoterol	Dec 1996	644	8.90×10^-2^	0.948	16.75
Tiotropium*	Sep 2002	748	3.537	0.969	8.50
Montelukast*	Sep 2002	78	4.82	0.969	7.08
**Sensory organs**					
Latanoprost*	Dec 1997	689	2.99×10^9^	0.923	7.92

To determine if the type of condition or therapeutic group had an influence over the rate of adoption, we tested for differences in the adoption times between Therapeutic subgroups (according to the Anatomical Therapeutic Chemical classification). Across the 10 therapeutic subgroups that included four or more drugs from our set (for a total of 67 medicines), no significant differences were found in the adoption times using an analysis of variance by ranks (p = 0.19).

The drugs were grouped according to those in which external forces were predominant (for 19 drugs, values of *p* were higher than the values of *q*, indicated by an asterisk in Table 1) and those in which the forces were evenly distributed or predominantly driven by internal forces (84 drugs). Under an analysis of variance by rank, the larger group of drugs, in which internal forces appeared to be dominant, was found to have significantly shorter adoption times (p = 0.02).

### Statins as an example of drug class adoptions

In Australia, HMG CoA reductase inhibitors (statins) were prescribed around 1.4 million times every month. There has been a rapid expansion of the market for cholesterol-lowering drugs, more than ten times the rate of prescription in 1992, which may be attributed to increased prevalence, increased diagnosis and increased marketing. New statins do not appear to subsume market share although simvastatin, fluvastatin and pravastatin have decreased in volume since the introduction of rosuvastatin. In 2009, the two predominant and increasing statins in the market were atorvastatin and rosuvastatin, which were first prescribed under subsidy in September 1997 and June 2006, respectively. The individual growth in prescriptions for all four of the statins introduced since 1996 conform to the pattern of diffusive adoption that appears to be common across the majority of drugs prescribed in Australia (s 3). The rate of adoption across the group does not match the order of entry or the maximum monthly prescription volumes. The lack of an obvious pattern is consistent with other drug classes in the study, and with a previous study on order of entry [46].

**Figure 3:**
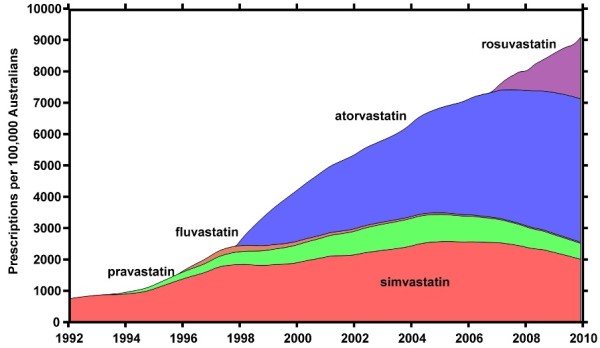
**The prescribing patterns of statins in Australia.** Cumulative prescription volumes for the four statins in the study (pravastatin, fluvastatin, atorvastatin and rosuvastatin), and prescription volumes for simvastatin, which was first prescribed under subsidy prior to 1992.

## Discussion

The results indicate that although the Bass diffusion model is capable of modelling adoption of new medicines in Australia, the adoption times of commonly-prescribed medicines are highly variable. The medicines in which internal forces were dominant in the adoption exhibited significantly faster adoption relative to their externally-dominant counterparts. However, the result should be interpreted with some caution. The internal/external divide does not appear to correspond to order of entry or the Anatomical main group or Therapeutic subgroup of the medicines in the sample. So while the Bass diffusion model suggests that two classes of adoption are present in the healthcare system (a result that also corresponds to current opinion, other markets and is exhibited at scale of the individual clinician), the result does not help to prospectively predict faster adoption of new medicines.

The limitations of the present study include the relatively small number of drugs in the anatomical groups, implying that an insignificant difference between groups may be a consequence of the sample size rather than an indication that the conditions or drug groups have little effect on overall adoption rates. Other limitations include the potential for bias associated with drugs that have not reached saturation – those predictions are likely to be less accurate regardless of how well the model fits for the available data. Other limitations specific to the mathematical modelling of adoption, both using the Bass diffusion model and more generally, are reported elsewhere [40,51-53].

The results show that internal forces such as social contagion are important factors affecting the adoption of new medicines. This finding is reflected in discussions around the perceptions of evidence [1], and studies demonstrating the presence of social contagion in the proliferation of evidence and opinion [54].

The effects of external forces such as the characteristics of medicines, competition, marketing effort and the dynamic production of evidence are considered as a single force in the Bass diffusion model. The median result for the time to saturation (8.2 years) suggests the presence of system inertia [55]. Fuchs and Milstein [2] provided a series of financial and social reasons for why clinicians and the organisations that support their decision-making are resistant to adopting cost-effective practices. It would be worthwhile modelling the different external factors explicitly in future studies.

## Conclusions

Alongside other models of adoption, the Bass diffusion model is capable of representing general adoption patterns for a broad range of medicines introduced and subsidised in Australia. The model estimates the contributions of internal and external factors that drive adoption and separate adoption patterns into two distinct categories. The wide range of adoption times revealed, and the lack of simple predictors to explain this variance, suggest that factors other than condition/class and order of entry affect a healthcare system’s response to the introduction of new medicines. Factors that are not considered in the model that may contribute to the variability include competition between interventions in the same class, the relative strength of marketing, and the effects of a highly dynamic evidence-base supporting the comparative effectiveness of medicines in each class.

The presence of system inertia suggests that the flow of new evidence into practice, and the rate of change of prescribing practices are important factors in determining how closely clinical decision-making reflects current perceptions of comparative effectiveness and safety. As a consequence, further research in the area would benefit from considering explicit links between the micro-scale of individual clinical decision-making and perceptions of evidence, the meso-scale of social contagion and marketing, and the macro-scale of regulation and competition.

## Competing interests

The authors declare that they have no competing interests.

## Authors’ contributions

AD conceived of the study, performed the analysis, and drafted the manuscript. BG and EC participated in the conception, analysis and coordination of the study, and contributed to critical revisions of the manuscript. JB, WR and RD participated in the conception of the study and contributed to the critical revisions of the manuscript. All authors read and approved the final manuscript.

## Pre-publication history

The pre-publication history for this paper can be accessed here:

http://www.biomedcentral.com/1472-6963/12/248/prepub
